# Binocular rivalry in autistic and socially anxious adults

**DOI:** 10.3389/fpsyt.2023.1181797

**Published:** 2023-06-28

**Authors:** Sarah Kamhout, Joshua M. Olivier, Jarom Morris, Hayden R. Brimhall, Braeden L. Black, Terisa P. Gabrielsen, Mikle South, Rebecca A. Lundwall, Jared A. Nielsen

**Affiliations:** ^1^Neuroscience Center, Brigham Young University, Provo, UT, United States; ^2^Department of Psychology, Brigham Young University, Provo, UT, United States; ^3^Department of Counseling Psychology and Special Education, Brigham Young University, Provo, UT, United States; ^4^Department of Psychiatry and Behavioral Sciences, Emory University School of Medicine, Atlanta, GA, United States

**Keywords:** binocular rivalry, autism, social anxiety, social anxiousness, switch rates

## Abstract

**Background:**

Social anxiousness is a pervasive symptom in both social anxiety disorder and autism spectrum conditions. Binocular rivalry, which occurs when different images are presented to each eye, has been used to explore how visual and cognitive processing differs across various clinical diagnoses. Previous studies have separately explored whether individuals with autism or anxiety experience binocular rivalry in ways that are different from neurotypical individuals.

**Methods:**

We applied rivalry paradigms that are similar to those used in previous studies of autism and general anxiety to individuals experiencing symptoms of social anxiousness at clinical or subclinical levels. We also incorporated rivalrous stimuli featuring neutral and emotional facial valances to explore potential overlap of social processing components in social anxiety and autism.

**Results:**

We hypothesized that higher levels of social anxiousness would increase binocular rivalry switch rates and that higher levels of autistic traits would decrease switch rates. However, stimulus condition did not affect switch rates in either diagnostic group, and switch rate was not significantly predictive of dimensional measures of either autism or social anxiety.

**Discussion:**

This may suggest a common mechanism for atypical visual cognition styles previously associated with social anxiety and autism. Alternatively, differences in switch rates may only emerge at higher trait levels than reported by the participants in our studies. Furthermore, these findings may be influenced by sex differences in our unique sample.

## Introduction

Social anxiety, defined as concern or avoidance of social situations in response to a fear of negative evaluation by others, can cause significant disruption to everyday routines and sense of well-being. For example, due to the social nature of daily living demands in many communities, social anxiety is associated with greater barriers to employment, including less educational attainment, less work experience, fewer job-specific skills, and fewer skills that involve human interaction in the workplace (1). Individuals with social anxiety are more than twice as likely to be unemployed or underemployed compared to those diagnosed with major depressive disorder, non-social anxiety disorders, or alcohol dependence (2, 3). Success in forming romantic relationships (4, 5) as well as adaptive friendships (6) is likely to be affected. Social Anxiety Disorder (SAD) is diagnosed when anxiety significantly disrupts daily function, with a prevalence rate estimated at 6.80% of American adults (7). Furthermore, up to 20% of the general population experiences subclinical levels of social anxiety symptoms, which may still be detrimental to function and quality of life (8–10).

Social anxiety often co-occurs with other diagnostic profiles. For example, up to 50% of autistic^1^ adults in the United States also experience significant social anxiousness^2^ associated with decreased success in everyday endeavors and additional obstacles to quality of life (12–14). While social anxiety and autism have distinct clinical profiles, one potential area of overlap is the link between social information processing and sensory processing, including early visual processing. Social anxiety disorder and autism are both linked with atypical sensory experiences (15, 16). Higher levels of social anxiety are associated with higher levels of sensory hypersensitivity in autistic and neurotypical participants (17, 18), and sensory hypersensitivity has been found to mediate the relationship between socially anxious traits and autistic traits (18). This finding suggests that part of the difficulty autistics face in social interactions is related to the increased sensory load inherent to social settings rather than the social nature of the exchanges themselves (18). Within the subgroup of autistic females, sensory traits do not fade over time and persist into adulthood (19). Tsuji et al. (20) also found sensory sensitivities persisting into adulthood to be associated with more internalizing disorders in autistic females (20).

Social anxiety disorder has been similarly associated with hypervigilance (i.e., increased attunement to certain sensations), especially in the presence of stimuli deemed threatening. Such sensory sensitivity has been linked to harm avoidance and agoraphobic tendencies in social anxiety disorder (21). For example, a recent eye-tracking study found that when experiencing anxiety, individuals diagnosed with social anxiety disorder demonstrated increased scan path lengths compared to a non-anxious comparison group. However, this same scanning effect was not present under emotionally neutral conditions (22). The authors theorized that this longer fixation path denoted less attentional control under threatening conditions, which inhibited effective environmental search. Electroencephalography has also shown that individuals with high levels of social anxiety experience greater sustained amplitude enhancement in early visual cortices associated with face-evoked signals than do low-social-anxiety controls (23).

In co-occurring autism and SAD, differences in sensation and perception may be related to shifts in global versus local processing. One study of autistic children performing a block design task found that the relationship between local processing and social skills was significantly moderated by anxiety levels (24). More dominant local-level processing may be in line with the Weak Central Coherence Theory of autism which suggests that individuals with autism show a greater preference for details rather than overarching themes of stimuli (25). A meta-analysis suggests that preference for local stimuli in autism might be related to unpruned synapses (25). The authors speculate that this reduced neuronal attenuation may explain the correlation between heavier brains and the likelihood of autism diagnosis. This phenomenon may result from decreased top-down modulation within early visual systems, leading to more intense sensory inputs than those experienced by neurotypical individuals (25). While local processing has yet to be thoroughly studied in social anxiety specifically, Hagenaars et al. (26) found that greater fixation on local-level inputs is associated with more intense recall and greater fear in those with anxiety-related to trauma and post-traumatic stress disorder (PTSD) (26). This finding may be suggestive of a role in threat response.

Binocular rivalry tasks may be especially well suited to explore similarities and differences in visual cognition associated with social difficulties in autism and social anxiety, given their ability to simultaneously assess local/global processing, threat bias, and sensory sensitivity. As individuals with autism or SAD can find verbal reports challenging, another benefit of utilizing binocular rivalry tasks is that they are a non-verbal measure of visual and cognitive processing (27, 28).

Binocular rivalry paradigms focus on the brain’s process for resolving differences in images presented to each eye. In day-to-day visual processing, resolving minor differences associated with eye position is common and automatic. Binocular rivalry tasks explore what occurs when the images presented to each eye are so distinct that they cannot be merged. During an experimental trial, individuals generally perceive rhythmic switching between the left eye’s and the right eye’s image. The rates at which this switching occurs seem to depend on stimulus conditions, with the visual system sometimes prioritizing one image over the other so that one percept is available for longer processing.

At the same time that competition in areas associated with early visual processing is necessary for switching to begin, altered inhibition and excitation associated with rivalry can also be observed in higher cortical structures (29, 30). While there is debate around the reason for binocular rivalry oscillations, or in other words, how rivalry phenomena may be related to extrinsic traits and symptoms, researchers theorize that perceptual switching results from variance in inhibition and excitation throughout the attention and visual systems (31, 32) and is especially modulated by GABA levels in the visual cortex (32). Computational models have similarly linked reduced inhibition with longer alternation rates (33) as well as with increased dysmetria and hypometria previously associated with autism spectrum conditions (34). Many genetic, postmortem, animal-model, and manipulation studies indicate that modulation of inhibitory and excitatory processes may also be a key component of other neural differences observed in autism (31, 35–37). Similar findings have also been mirrored in subjects with social anxiety (35) and anxiety generally (36–40). However, these conditions are rarely studied in tandem or with more than one stimulus type at a time.

Using rivalrous images of gratings stimuli, Nagamine and colleagues demonstrated that binocular rivalry induced higher switch rates in a high anxiety group (38). This faster switching may indicate that those with higher anxiety experience more perceptual competition between the two competing images and potentially less sensory suppression (38). Using similar geometric stimuli, Wykes et al. (39) found that neurotypical young adults with more autistic traits experienced slower switching than subjects with fewer autistic traits (39). However, this correlation was not present when more complex images of objects were used. This lack of effect is consistent with the findings of Said et al. (40) who found that binocular rivalry with geometric images did not reliably differentiate high-functioning autistic adults from control participants and did not predict symptom severity (40). It is also consistent with the findings of Karaminis et al. (41) who found that neither rivalrous geometric nor rivalrous object images elicited significantly different switch rates in autistic children as opposed to same-age controls (41). On the other hand, Robertson and colleagues found that when viewing rivalrous images of objects, autistic adults experienced longer periods of unresolved perceptions between switches, which resulted in slower switch rates (31). Additionally, the duration of mixed perceptual states was positively correlated with increased levels of autistic traits, while switch rate was negatively correlated with autistic traits. These findings have also been replicated using object images, verified with electroencephalography, and associated with changes in neurotransmitter levels as measured by magnetic resonance spectroscopy, with slower switch rates and more persistent mixed percepts predicting participants’ diagnostic status with 87% accuracy (37, 42).

When specifically interested in social symptoms, researchers often prefer studying responses to faces (e.g., expressing hostility or friendliness) in socially anxious versus non-socially anxious individuals. The usual hypothesis is that there will be greater performance differences in threatening versus non-threatening faces for individuals with social anxiety than individuals without social anxiety. Several studies using methods other than binocular rivalry demonstrate these performance differences for individuals with social anxiety disorder (43–46). Similar findings are also available for autistics. For example, in an emotion recognition task using electroencephalography, autistic boys displayed significantly reduced neural responses to negatively valenced (angry and fearful) faces compared to neurotypical controls (47). Another study, in which participants identified whether facial expressions were congruent or incongruent with an image’s body language, found that while autistics had shorter viewing times than controls overall, they were especially avoidant of images depicting fear (48). Interestingly, in this and other congruency paradigms, autistic participants were also less accurate than controls at differentiating fearful from angry faces (48–50).

While binocular rivalry studies in general anxiety have found that participants reporting higher levels of anxiousness experience accelerated switch rates (38), binocular rivalry studies in individuals with social anxiety are less common. These studies primarily focus on initial percepts, or the first rivalrous image a participant reports seeing, rather than ongoing switching rates. They indicate that binocular rivalry may be sensitive to social threat, with individuals with social anxiety seeing the threatening face first more often than individuals without social anxiety (51, 52).

The overall aim of this study was to utilize binocular rivalry paradigms to examine similarities and differences in switch rates in response to various stimulus conditions among three groups of adults: adults with a diagnosis of autism or high levels of autistic traits, adults with a diagnosis of SAD or high levels of socially anxious traits (both without an autism diagnosis), and non-anxious neurotypical adults. Given our focus on social anxiousness, we also integrated novel facial stimuli. Our specific hypotheses, based on the results from previous studies (31, 37, 38, 42), were as follows:

1. Across groups, switch rates would be different for different facial valences
2. Switch rates would predict diagnostic group assignment
  - Higher levels of socially anxious traits would predict faster switch rates.
  - Higher levels of autistic traits would predict slower switch rates.
3. Predictions for Hypotheses 2a and 2b would be more clearly observed in facial vs. geometric stimuli

## Materials and methods

### Participants

This study combined data from three studies conducted at Brigham Young University that utilized the same binocular rivalry task and recruited participants for traits related to autism or social anxiety as well as non-anxious neurotypical controls. All studies were approved by the sponsoring institution’s IRB (F19260, F2020-242, and IRB2020-429) and funded through internal seed grants and family foundations.

The clinical groups were recruited as a follow-up to previous studies of mental health in women who find social situations confusing or exhausting (53, 54). This sample included women diagnosed with autism who were recruited from existing research databases, local assessment clinics, and via word-of-mouth, and socially anxious women who were recruited from local university and community counseling clinics and via word-of-mouth. All participants were paid for their time or were invited via the university research recruitment system where students were given course credit for participating.

In all, 223 adults (*M* Age = 21.9 years ±3.44, *R* = 18–44 years, Table 1) participated in the binocular rivalry tasks, with 47 representing clinically significant autistic traits. We focused on adults given previous studies’ significant observations in this age group (31, 37, 38, 42) as opposed to contradictory findings in pediatric cohorts (41). In order to maximize spectra of symptom presentation, we did not match participants based on age, but instead controlled for age in secondary analyses (see Supplementary Table S4). Approximately half of these participants were identified by clinical diagnosis outside of the study (*n* = 6), internal clinician judgment (*n* = 4), or clinician administration of the *Autism Diagnostic Observation Schedule-2nd Edition (ADOS-2), Module 4* (55) (*n* = 14). In order to account for a wide range of symptom presentations, we included all participants with moderate or high ADOS-2 evidence of autism in the autistic group. We used the lower part of that threshold (total score of 5) for females since previous studies have suggested they may score lower on the ADOS-2 even when experiencing symptoms at similar severity levels to males (56, 57). All participating males assigned to the autism group had a total score of 6 or higher.

**Table 1:** Participants by diagnostic group.

	*N*	Age	Sex F/M (%F)	Psychotropic Medication Y/N (%Y)
ASC	47	23.72 ± 5.14 [18–44]	39/7 (84.8%)	17/25 (40.5%)
SA	25	22.16 ± 2.04 [19–27]	21/4 (84.0%)	19/3 (86.4%)
NT	151	21.21 ± 2.57 [18–35]	98/49 (66.7%)	1/33 (2.9%)
Total	223	21.9 ± 3.44 [18–44]	158/60 (72.5%)	37/61 (37.8%)

As we combined data from several studies, screening questions varied slightly between recruitment groups. The number of participants that provided responses for each variable are as follows: (1) Age: *N* = 203, (2) Sex: *N* = 218, (3) Psychotropic Medication: *N* = 98.

An additional 23 participants who were not clinically tested were also included in the autistic group due to self-report of clinically relevant symptoms at or near established cutoffs for the *Autism Spectrum Quotient (AQ)* (58) (*n* = 19) (≥26) or the *Ritvo Autism Asperger Diagnostic Scale-Revised (RAADS-R)* (59) (*n* = 4) (≥65). Twenty-five participants exhibited clinically significant socially anxious traits according to clinician evaluation of the *Mini International Neuropsychiatric Interview (MINI)* interview (60) and below-threshold ADOS-2 scores, and 151 demonstrated neurotypical or subclinical levels of these traits according to each study’s measures.

As one of the studies included here specifically included only women, our sample was primarily female (72.5%), even within the high autistic trait group (84.8%), where women have been historically underrepresented (61, 62). Across the 158 participants who reported their ethnicity, 87.3% identified as white, 12.7% identified as Hispanic or Latino, 4.4% identified as Asian, 1.3% identified as African American or Black, and 5.1% identified as Pacific Islander, Native American, or other.

### Descriptives

**Figure 1:**
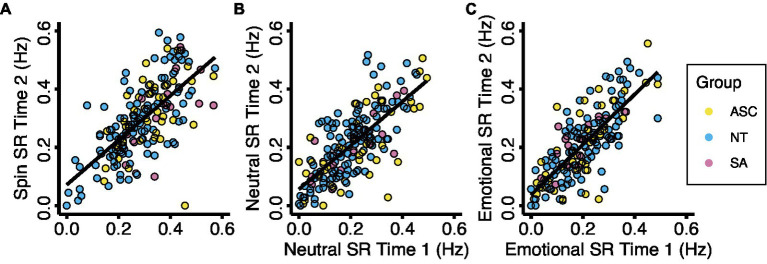
Test-retest reliability for the three experimental conditions. Each data point represents the mean binocular rivalry switch rate for one participant on two study visits during each experimental condition. Results show a significant correlation for **(A)** the spin condition (ICC = 0.806, F(188, 189) = 5.178, *p* < 0.001), **(B)** the neutral condition (ICC = 0.846, F(188, 188) = 6.483, *p* < 0.001), and **(C)** the emotional condition (ICC = 0.867, F(183, 184) = 8.073, *p* < 0.001). Abbreviations: ASC, autism spectrum condition; NT, neurotypical; SA, social anxiety; SR, switch rate.

No demographic factors, measure scores, or switch rates exhibited significant skew. We removed outliers above or below 1.5 times the interquartile range for each measure and switch rate condition (Supplementary Table S1). Using the consistency definition of intraclass correlation (ICC) and a 95% confidence interval, we found good reliability between the switch rates measured in sessions 1 and 2 (Spin ICC = 0.806, *F*[188, 188] = 5.153, *p* < 0.001; Neutral ICC = 0.845, *F*[187, 187] = 6.464, *p* < 0.001; Emotional ICC = 0.877, *F*[183,183] = 8.133, *p* < 0.001; see Figure 1).

Depending on their assigned study, some participants took the AQ more than once, in which case scores for both administrations were averaged. One participant’s data was omitted from analysis entirely due to a variance of 32 points in AQ score between sessions and only one trial of binocular rivalry having codable time stamps.

As we combined binocular rivalry data from three studies, participants completed several measures of autistic and socially anxious traits. Table 2 describes participant group scores for the behavioral trait measures. The average AQ score in the autism group was above the threshold of 26 for clinical referral. The social anxiety group did not exhibit clinically significant AQ scores (*M* = 21.90, *SD* = 6.33). While many participants met standard criteria for social anxiety and also exhibited clinically significant total LSAS scores, the average total LSAS score for all groups was below the clinical threshold of 90 (ASC *M* = 63.1 ± 12.04, SA *M* = 72.5 ± 11.27), which may suggest a comprehensive range of symptom severities recruited in each group and effective subclinical representation. Average total LSAS scores in the neurotypical group were approximately half that of those in the autism and social anxiety groups (*M* = 32.28 ± 7.58).

**Table 2:** Psychometric scores.

	ASC	SA	NT	Total
AQ	30.4 ± 6.13	21.90 ± 6.33	14.90 ± 4.77	18.80 ± 8.14 [6–40]
RAADS-R	120.73 ± 37.30	--	30.64 ± 11.16	90.67 ± 53.02 [15–178]
TCI-HA	76.04 ± 17.7	--	54.22 ± 13.82	57.95 ± 17.00 [30–94]
LSAS-Fear	36.80 ± 13.31	42.50 ± 13.27	17.14 ± 7.60	32.67 ± 15.85 [2–62]
LSAS-Avoidance	26.30 ± 10.77	30.00 ± 9.26	15.14 ± 7.56	24.10 ± 11.11 [2–45.5]
LSAS-Total	63.10 ± 23.80	71.53 ± 22.73	31.19 ± 15.95	56.10 ± 27.08 [4–108]

### Binocular rivalry equipment setup and task

**Figure 2:**
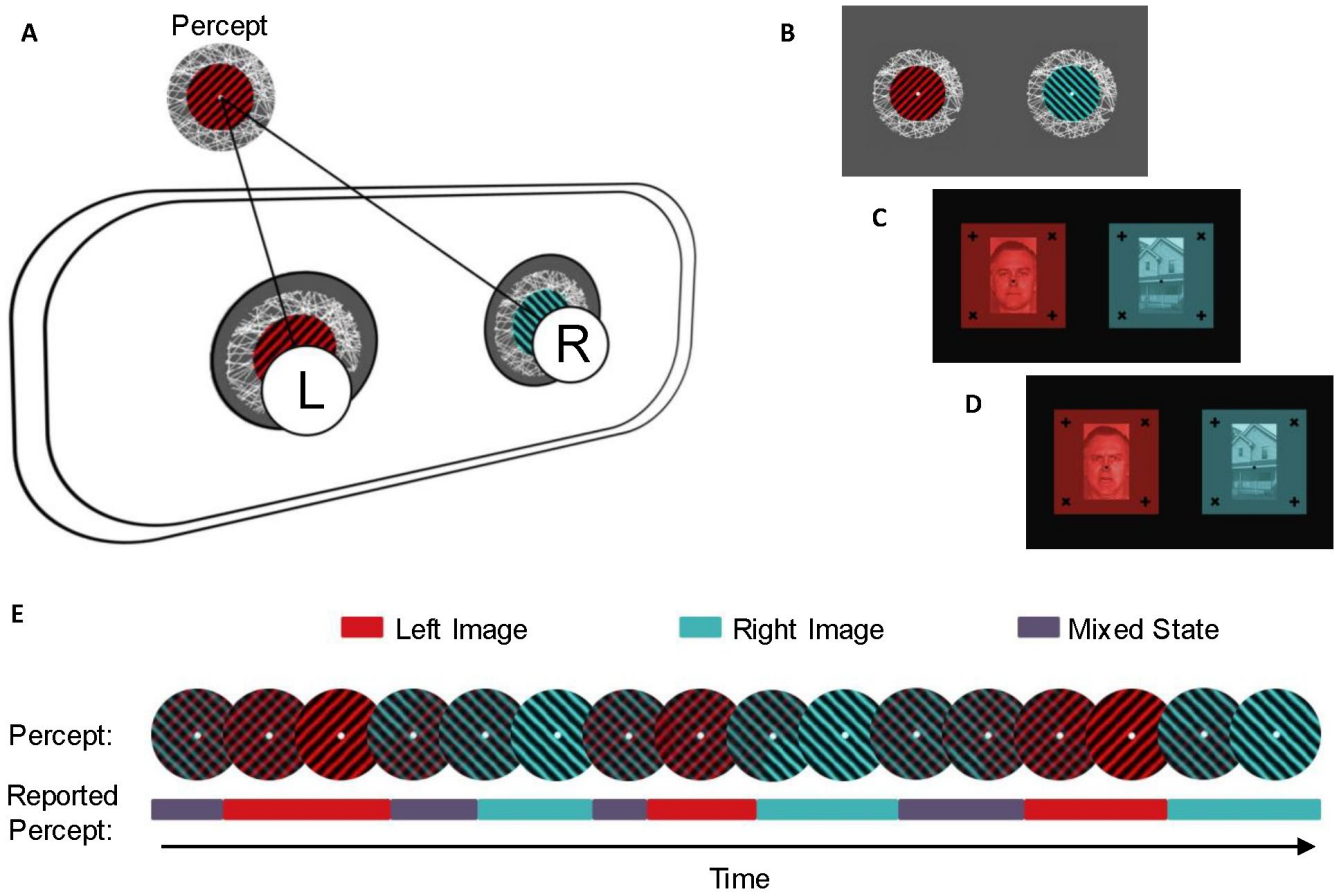
Experimental setup and stimuli. **(A)** Stimuli were presented dichoptically through a virtual reality headset. The left eye (L) and right eye (R) were simultaneously presented with distinct images that were visually aligned using centered fixation points, so the left and the right image would be seen as a single percept. **(B)** Stimuli for the spin condition. Each stimulus was presented with an identical fixation point and textured border and animated to rotate clockwise to enhance stable vergence. The colored grating on the left stimulus was aligned perpendicular to the colored grating on the right stimulus. Stimuli for the neutral **(C)** and emotional **(D)** conditions were presented with identical fixation points and solid frames. The left stimulus was a red monochrome image of a person with an emotionless or emotional facial expression, while the right stimulus was a blue monochrome image of a house. **(E)** Example of perceptual switches and reported percepts during the binocular rivalry task. During binocular rivalry, the dominant percept alternates between the dominant perception of the left image (red), the dominant perception of the right image (blue), and a mixed percept (purple). Participants reported perceptual switches in real-time using keypresses.

All three studies utilized the same Binocular Rivalry task. To measure the binocular rivalry switch rate, we used a 7,140–79 LEEDS Luxury Virtual Reality Headset, which holds a smartphone inside (Figure 2A). We displayed three images to each participant, each representing a different binocular rivalry condition (Figures 2B–D). The first rivalrous image subjects viewed included two monocular images adapted from stimuli used in Sandberg et al. (63): a red circular patch of square wave gratings presented to the left eye and a blue circular patch of square wave gratings presented to the right eye (spatial frequency = 3 cycles/degree, contrast = 100%). Due to difficulties with achieving stable vergence and minimizing piecemeal rivalry while viewing this stimulus with the virtual reality headset, both patches were made to rotate continuously in a clockwise direction at a rate of 0.33 rotations/s so that the gratings on the red circular patch were always perpendicular to the gratings on the blue circular patch (64, 65).

We also displayed a composite image of a face with an emotional facial expression (angry; presented to the left eye) and an image of a house (presented to the right eye). The third image composite was an image of a face with a neutral facial expression (presented to the left eye) and an image of a house (presented to the right eye). We selected these facial valences based on previous research which compared response to threatening (angry and/or fearful) faces with neutral faces (66). In particular, a metanalysis of visual paradigms found that increased anxiety is linked with more frequent orientation to threatening images as compared with neutral ones (67). In binocular rivalry tasks, emotional faces have been shown to predominate over neutral ones (68). In an autistic sample, Van der Donck et al. (47) had also identified similar neural stimulation in happy, sad, and neutral facial conditions (47). In order to maximize potential effects when pairing faces with a neutral object in the classic face-house design (69), we opted to use one neutral face and one angry face, theorizing that a neutral face would be most affectively congruent with the neutral house, and a face depicting an angry valence well-established to be interpreted as threatening would be least congruent. Face images were obtained from the Pictures of Facial Affect collection by the Paul Ekman Group (70). All images included a fixation point and a textured background or nonius lines to maintain stable vergence (64). Participants used a LabView program on a desktop computer and the arrow keys on a keyboard to record when their perception changed from seeing one image as dominant to the other.

Participants were presented with up to 10 versions of each image set to determine which version allowed for the best visual alignment. Each version contained identical images from the respective stimulus condition with varying distances between central fixation points. After the six trial runs for the first image set were complete, participants were instructed to remove the goggles to begin the fitting procedure for the following image.

Participants were shown one of the three image sets for six 30-s intervals. During each interval, participants were instructed to self-report perceptual switches between the image presented to the left eye, the image presented to the right eye, or a mixed percept (defined as less than 80% dominance) using arrow keys on a keyboard (Figure 2E). After the 30 s were finished, subjects were told to close their eyes and rest for 15 s, after which they would open their eyes again and continue the next 30-s reporting period. This process was repeated for each participant for the other two image sets.

In order to ensure the accuracy of keyboard reporting and to account for changes in focus upon ocular relaxation, participants were given two practice rounds before the first geometric image set (spin) and one practice round before each subsequent set (emotional or neutral). After each practice run, the researcher vocally confirmed with the participant that the image remained clear and comfortable to view.

If participants completed both the first and second binocular rivalry appointments in 1 day, they were required to rest for at least 10 min between segments one and two, during which time they were encouraged to relax their eyes by looking at least 5 feet ahead.

### Behavioral trait measures

Several self-report surveys and diagnostic measures were acquired, although not all participants completed every measure. There were slightly different combinations of behavioral measures; the total *n* collected for each measure is identified in the description. All surveys were administered via the Qualtrics online software platform (Qualtrics, Provo, UT).

#### Autism symptoms

##### Autism spectrum quotient (AQ; *n* = 217)

This 50-question survey (58) is frequently used to quantify autism-associated traits in clinical and neurotypical cohorts. Previous studies have found it to have internal reliability above 0.7 (71) and high test–retest reliability (72).

##### Autism diagnostic observation schedule-2nd edition, module 4 (ADOS-2, *n* = 35, Mod 4)

This diagnostic assessment (55) was completed by 35 individuals in the sub-study with the highest concentration of autism traits. Together with the MINI, it was used to differentiate between participants with social anxiousness related to autism or that which was more reflective of an independent social anxiety disorder. Many researchers and clinicians consider the ADOS-2 (55) the gold standard for observing traits of autism. It involves targeted conversation and activities to press for reciprocal social interaction. It was administered by trained clinical researchers.

#### Anxiety symptoms

##### Temperament and character inventory, harm avoidance scale (TCI-HA; *n* = 152)

The TCI-HA (73) was given to assess anxious traits, which may conflate autism and social or generalized anxiety diagnosis and possibly affect binocular rivalry switch rates. This measure of anxiety is of particular interest since it is used to assess traits of many severities, including subclinical levels of anxiety which are significant but may be overlooked by more strictly clinical measures.

##### Liebowitz social anxiety scale (LSAS; *n* = 57)

This survey (74) is used in the assessment of SAD symptoms. It correlates with the Social Phobia Scale and the Social Interaction Anxiety Scale (75) and scores are also associated with overall fear levels (76). In the first half, participants use a Likert scale of 0–3 to rank the fear they would feel if engaged in various social situations or interactions. Then, they rank the same scenarios based on how much they would avoid such an encounter. Its ability to capture both internal reactions and external behaviors is valuable to understanding potential masking of anxiety symptoms.

#### Additional measures

##### Demographics questions

These varied across study and included questions about age (*n* = 203), biological sex (*n* = 228), race and ethnicity (*n* = 157), handedness (*n* = 91), and psychotropic medication usage (*n* = 98). We also collected information on adverse childhood experiences, PTSD symptoms, suicidality, sleep, health concerns, diet, and exercise that were part of a more extensive study and not intended for this analysis of binocular rivalry data.

### Data analysis

To calculate binocular rivalry switch rates, we counted the number of times participants indicated a shift between left-dominant and right-dominant perceptions and divided this count by the 30 s in which keypresses were collected during each trial in order to find the number of switches per second. We did not count partial switches (mixed percepts), which were reported via an up arrow key press.

Initially, we measured the test–retest reliability of our binocular rivalry protocol by calculating intraclass correlation coefficients for each subject’s average perceptual switch rate for each condition (spin, neutral, and emotional) in session one and that of the same condition as measured during session two (Figure 1). Once reliability was confirmed, corresponding switch rates from the two repeated sessions were averaged before analysis.

To examine our first hypothesis regarding the effect of stimulus conditions (spin, neutral, or emotional) on switch rate, we used a one-way ANOVA to compare switch rates between image valences. Then, based on that test’s significance, we completed post-hoc t-tests to isolate which stimulus conditions were driving the effect. Each compared the effect of two of the three stimulus conditions (spin and neutral, spin and emotional, or neutral and emotional) on switch rate.

Next, we examined our second hypothesis, namely, whether any image condition(s) could predict diagnostic group assignment. We used a similar omnibus ANOVA to examine our second hypothesis regarding whether any image condition(s) could predict diagnostic group assignment. Since the stimulus condition effect could potentially overshadow the diagnostic group effect in the omnibus test, we repeated the ANOVA model three times, once for each image valence (spin, neutral, or emotional), to compare the effect of the diagnostic group on switch rates for each condition. Finally, in order to assess differences in performance in autism, social anxiety, and neurotypical groups within any significant image condition(s), if applicable, we planned to conduct three additional pairwise regressions with switch rate data from two diagnostic groups within one image valence at a time (Spin ASC vs. Spin NT, Spin ASC vs. Spin SA, or Spin SA vs. Spin NT).

Due to unequal sample sizes and potential unequal variance between diagnostic groups, we also used the non-parametric Kruskal–Wallis test to further assess diagnostic group differences. As in our previous ANOVA model, since any stimulus condition effects identified for Hypothesis 1 could potentially overshadow any diagnostic group effect in the omnibus test for Hypothesis 2, we also compared the effect of diagnostic group on switch rates for each condition by repeating the Kruskal–Wallis test three times with data from only one image valence (spin, neutral, or emotional) in each iteration. In parallel form, we also planned to compare differences in performance in autism, social anxiety, and neurotypical groups within image condition(s) via three follow-up Brunner-Munzel tests with switch rates from two diagnostic groups within one image valence at a time (Spin ASC vs. Spin NT, Spin ASC vs. Spin SA, or Spin SA vs. Spin NT).

As the availability of demographic information such as age, sex, and psychotropic medication varied according to the study in which subjects participated (Table 1), we removed covariates from our original analyses to maximize power. In order to verify that any observed effects were due to diagnostic group or stimulus condition, we repeated each post-hoc analysis with age, sex, and medication usage included. We also repeated the analyses using only data from participants with clinician-verified ADOS-2 scores (35 participants) or, in the absence of available ADOS-2 scores, report of non-study clinical diagnosis of autism (6 participants) rather than relying on thresholds assigned by self-report measures (AQ, RAADS-R, or TCI). To address potential experimental confounds, we repeated the analyses described above using only participants who had data from both time points, as some were only able to complete one session.

Following our analysis of differences between stimulus types and diagnostic groups, we also examined the effect of more broad symptom presentations on switch rates by performing a series of separate regressions with either AQ, TCI-HA, Liebowitz Total, or ADOS-2 scores as the outcome and with switch rate for one stimulus category, age, sex, and the number of data points as predictors. Each regression for each symptom measure was repeated three times, once with switch rates specific to each stimulus condition, for a total of 15 tests. We corrected for multiple comparisons using the Holm–Bonferroni Sequential Correction for each hypothesis question (77).

## Results

### Main analysis

The aim of this study was to investigate differences in binocular rivalry dynamics among adults with a diagnosis of autism or high levels of autistic traits, adults with a diagnosis of SAD or high levels of socially anxious traits, and non-anxious neurotypical adults. First, we hypothesized that across groups, binocular rivalry switch rates would be different for the two facial stimuli. In order to discern the possible effects of the image type on switch rate, we conducted a one-way ANOVA. Condition was a significant predictor of switch rate (*F*[2, 652] = 28.152*, p* < 0.001). We completed three follow-up paired t-tests to isolate which experiment conditions drove the effect and investigate whether any effects were most prominent within the facial conditions. Image condition was predictive of switch rate for neutral versus spin (*F*[1, 437] = 66.75, *p’* < 0.001) and emotional versus spin (*F*[1, 434] = 76.92, *p’* < 0.001), but not for emotional versus neutral (*F*[1, 433] = 0.344, *p’* = 0.558). Therefore, the spin condition drove the effect of condition rather than differences in facial valence.

**Figure 3:**
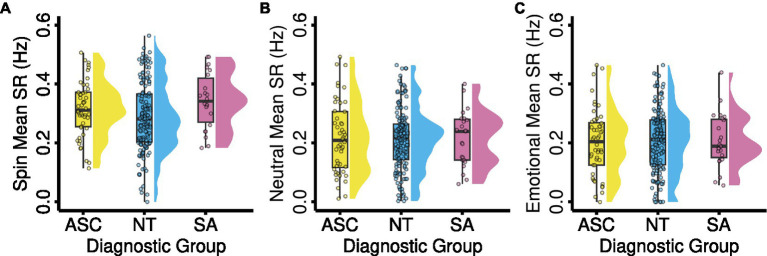
Mean binocular rivalry switch rate by diagnostic group. Mean switch rates for ASC, NT, and SA diagnostic groups for **(A)** the spin condition, **(B)** the neutral condition, and **(C)** the emotional condition. Abbreviations: ASC, autism spectrum condition; NT, neurotypical; SA, social anxiety; SR, switch rate.

Second, we predicted that switch rates would be predictive of diagnostic group, in that an increasing number of socially anxious traits would positively correlate with switch rate and an increasing number of autistic traits would negatively correlate with switch rate. Although diagnostic group assignments aligned with established dimensional measures of autism and social anxiousness, the range of our observed switch rates remained largely consistent between groups. This more narrow range of switch rates also bore out between image conditions. While the spin condition had a slightly wider range of observed switch rates compared to the neutral or emotional facial conditions (Table 3), those measured in all image conditions and diagnostic groups showed as few as no oscillations per second and no greater than 0.564 oscillations per second. To examine whether there were diagnostic group differences in switch rates, we initially repeated the previous omnibus ANOVA but used the diagnostic group, rather than condition, as the predictor. While this was not significant (*F*[2, 652] = 0.445, *p* = 0.641), to ensure any effects of the diagnostic group were not overshadowed by the condition effect present due to including switch rates from all conditions in the model and to continue to explore our third hypothesis regarding whether effects were different between geometric and facial valences, we performed three planned pairwise regressions with each model examining possible group effects of switch rate within one stimulus condition. There were no significant group differences in switch rate by diagnostic group in any trial type (Spin: *F*[2, 217] = 2.43, *p’* = 0.271; Neutral: *F*[2, 216] = 0.183, *p’* = 1.00; Emotional: *F*[2, 213] = 0.155, *p’* = 1.00; see Figure 3).

**Table 3:** Average binocular rivalry switch rates (switches/s) by diagnostic group and condition.

	ASC	SA	NT	Total
Spin	0.316 ± 0.094 [0.114–0.506]	0.343 ± 0.095 [0.183–0.492]	0.287 ± 0.117 [0–0.564]	0.299 ± 0.111 [0–0.564]
Neutral	0.219 ± 0.122 [0.011–0.492]	0.216 ± 0.093 [0.061–0.400]	0.212 ± 0.105 [0.003–0.464]	0.214 ± 0.107 [0.003–0.492]
Emotional	0.202 ± 0.113 [0–0.453]	0.208 ± 0.092 [0.056–0.439]	0.210 ± 0.106 [0–0.464]	0.208 ± 0.106 [0–0.464]

In order to account for unequal sample sizes and unequal variance between controls and socially anxious participants within the spin condition, we performed a Kruskal–Wallis test with average switch rates for all conditions as the outcome and diagnostic group as the predictor. While this was not significant (*X^2^* [2, *N* = 652] = 1.03, *p’* = 0.597), in similar fashion to our previous model, we performed three follow-up Kruskal–Wallis tests with each examining possible diagnostic group effects on switch rates within one stimulus condition. Once more, for all stimulus conditions, there were no significant differences in switch rates according to diagnostic group (Spin: *X^2^* [2, *N* = 652] = 5.24, *p’* = 0.218; Neutral: *X^2^* [2, *N* = 652] = 0.21, *p’* = 1.00; Emotional: *X^2^* [2, *N* = 652] = 0.27, *p’* = 1.00; see Figure 3). Additionally, after correcting for multiple comparisons, follow-up Brunner–Munzel tests revealed no significant differences in switch rates between diagnostic groups (Supplementary Table S2), with no nonparametric findings diverging from those of the original parametric models.

**Figure 4:**
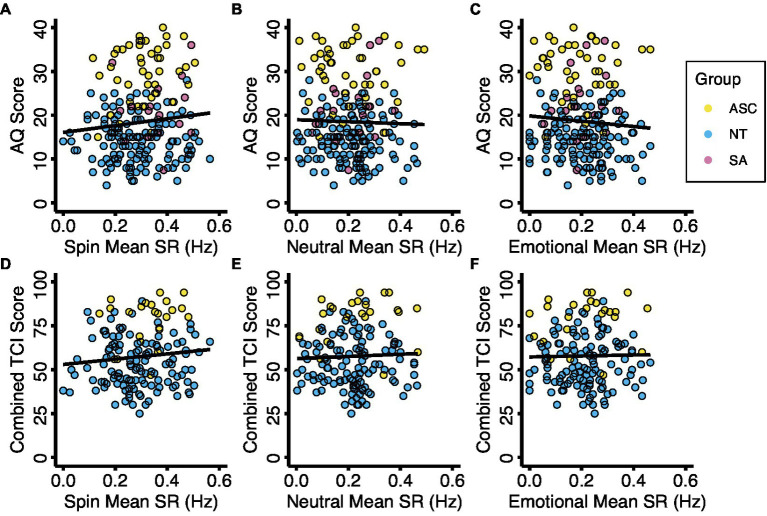
Binocular rivalry switch rate with autistic traits and anxious traits. Diagnostic groups are represented by color. Relationships between mean switch rates and autistic traits **(A–C)** and anxious traits **(D–F)** for the three experimental conditions. Abbreviations: ASC, autism spectrum condition; AQ, Autism Spectrum Quotient; NT, neurotypical; SA, social anxiety; SR, switch rate; TCI, Temperament and Character Inventory, Harm Avoidance Scale.

We also used multiple regression analyses to explore whether average binocular rivalry switch rates in any image condition were predictive of self-report measures of autistic or anxious traits. We controlled for age, sex, and the total number of trails administered. AQ, TCI, and Total LSAS scores were not significantly predictive of switch rate in spin, neutral, or emotional image valences (Figure 4; Supplementary Table S3).

### *Post hoc* analyses

Since this analysis was based on data collected through several different projects and some survey measures varied between them, we did not have handedness, age, or medication data for a significant number of our participants. Given that effect sizes of binocular rivalry switch rate differences may be small, in order to maximize power by maintaining maximum sample size, we did not include these factors in our original models. However, since other researchers have suggested that they may also influence binocular rivalry switch rates, we repeated our initial ANOVA models with these factors as well as sex included and found no significant differences in the overlying trends. Although age emerged as a consistent positive predictor across diagnostic groups and stimulus conditions (*F*[1, 287] = 17.419, *p* < 0.001), stimulus condition was still only predictive of switch rate when comparing geometric stimulus to facial stimuli, but not between facial valences (Emotional vs. Neutral: *F*[1, 191] = 0.206, *p’* = 1.00; Emotional vs. Spin: *F*[2, 216] = 0.183, *p’* = 1.00; Neutral vs. Spin: *F*[2, 213] = 0.155, *p’* = 1.00). (For a complete statistical report, see Supplementary Table S4).

We also repeated our original analyses of diagnostic group differences with only those participants whose group assignment had clinical confirmation (as opposed to using suggested cutoffs for self-report measures alone), which again did not reveal any significant differences in switch rate between ASC, NT, or SA participants (*F*[2, 547] = 0.197, *p* < 0.821; Supplementary Table S5).

Finally, we also added the number of trials each participant completed for each condition as a predictor since a small percentage of participants did not complete all 12 iterations due to visual fatigue or study attrition, but this was not significantly predictive in any model (*F*[1, 651] = 0.101, *p* < 0.751; Supplementary Table S6).

## Discussion

We did not replicate previously reported significant switch rate differences in autism, social anxiety, and neurotypical populations. First, we examined whether switch rates varied according to image type and found that while participants of all three groups responded differently to geometric as opposed to facial images, there were no significant differences in switch rates for angry versus neutral facial expressions. We also did not find significant differences in switch rate according to diagnostic group classification. Similarly, no switch rates observed during any image conditions were predictive of measures of social anxiousness and autistic traits.

While our results vary from that of Nagamine et al. (38) and Robertson et al. (31, 37), and Spiegal et al. (42) whose designs we modeled, they are in line with the lack of autism-associated changes in switch rates noted by Said et al. (40), Karaminis et al. (41), and Wykes et al. (39) (Object Stimuli). While binocular rivalry is still an emerging area of biomarker research, given that findings around slower switch rates in autism are the most robust of the three diagnostic groups we examined, we are particularly interested in our lack of effect for this cohort. As high rates of comorbid social anxiety are reflective of the autistic community at large, if anxiety supersedes foundational brain differences seen in autism or if symptoms of social anxiousness are representative of the same cognitive processes in both autistic and non-autistic socially anxious groups, our focus on socially anxious autistic individuals may have led to an overshadowing of previously observed binocular rivalry effects. Namely, anxiety’s proposed tendency to increase switch rates might counteract or overcome the slower switching associated with autism alone. Furthermore, sensory sensitivity has been suggested as a cause of heightened anxiety in autism, meaning that some autistic participants may experience exceptionally high anxiety levels during binocular rivalry tasks regardless of what stimuli are presented, which may illuminate the absence of effect of differing facial valances in this group (78).

Beyond the implications of potential comorbidity, our participant pool also had a significant female majority, even within the autism group. Since prior studies of autism utilizing binocular rivalry used primarily male samples and some researchers theorize that autism may be related to biological sex and gender differences in cognition, particularly as explained via the extreme male brain theory of autism and the female protective effect (19, 79), our inability to replicate the switch rate slowing previously observed in autistic groups may be related to emerging sex differences rather than a broader lack of autism-associated effects. It is also possible that, regardless of sex, the neural mechanisms behind social anxiousness in SAD and autism are so similar that differences are not measurable via binocular rivalry tasks. Finally, the associations between higher levels of sensory sensitivity and anxiety and masking or the effects of camouflaging abilities are not yet fully understood in autistic females, which warrants further exploration.

Concerning our lack of observed differences in social anxiety, 23 of our participants with SAD and 18 with autism were recruited through a more extensive examination of suicidality which is increasingly recognized as especially prevalent in autistics (80) and is also associated with depression. It has recently been shown that depression may slow switch rates (81). If binocular rivalry phenomena are less condition-specific than previously theorized, it is possible that a similar sort of slowing occurred among socially anxious participants in our sample who also experience depressive symptoms, with any social anxiety-associated increases in switch rate (38) being counteracted by comorbid depression’s associated decrease.

We also hypothesize that the lack of significant differences in switch rate between facial valences may be related to our choice of a neutral face as a control for the emotional one, which depicted anger. It is possible that individuals with social anxiety, especially those with autism, may subconsciously recognize neutral faces as threatening and, therefore, experience switch rates similar to those observed when viewing the angry expression more widely associated with threat bias. For example, in the still face paradigm, infants with a high genetic likelihood of having autism exhibit fewer prosocial behaviors when interacting with an unresponsive, neutral-faced caregiver, and these decreases in social bids corresponded to greater difficulties with emotional regulation later on in life (82). While this decrease in outreach could reflect a lack of interest, given that the high-likelihood infants still exhibited other signs of distress and frustration at similar levels to the typically developing group, it has been theorized that this withdrawal is a stress/freeze response resulting from heightened emotional and sensory sensitivity to the ambiguous response (82). This idea also aligns with Tottenham et al.’s (83) findings that autistic participants’ visual avoidance of neutral faces corresponded to the perceived threat level for each image (83). The same study also used functional magnetic resonance imaging to observe that while autistic individuals experienced differential amygdala activation for all facial valances, the effect was most strong for neutral stimuli. A similar study also found that during exposure to neutral faces, right amygdala activation was heightened in those with social anxiety disorder but not in controls (11). This observation may suggest a similar condition-associated tendency to assign negative valences to images that neurotypical participants process as non-threatening. Therefore, we suggest that future studies of binocular rivalry in socially anxious populations, especially those with a high prevalence of autism, incorporate a wider variety of facial expressions or utilize a less emotionally ambiguous facial affect such as happiness for baseline comparisons.

We identified three primary limitations in the present study. First, data collection relied on participant self-report of perceptual switches. Although participants were trained to respond to the task consistently and test–retest reliability was sound, the precise instance of a perceptual switch during binocular rivalry is ambiguous by nature. Additionally, the experimental paradigm did not account for potential individual differences in the onset of binocular rivalry (84). Third, our study did not explore associations between neural activity and self-reported switch rate, although prior evidence strongly suggests self-report accuracy in neurotypical and autistic individuals (42).

## Conclusion

Although we did not replicate the significant switch rate differences related to anxiety and autism that were observed in prior studies (31, 37–39, 42, 85), we did replicate the absence of a significant effect observed by other teams (39–41) and speculate that this lack of effect may be due to similar cognitive processes involved with social anxiousness in autism and social anxiety alike. We also investigated the external validity of previously observed binocular rivalry trends by incorporating a larger participant group than had been accessible during prior investigations and by representing a more comprehensive range of clinical and subclinical levels of social anxiousness and autistic traits. More broadly, we developed and verified a replicable binocular rivalry protocol utilizing virtual reality goggles in a sensory-sensitive group.

Our absence of observed visual processing differences according to facial emotional valence, as was previously measured with eye-tracking and congruency tasks, might illuminate future discussions of binocular rivalry as a measure of threat detection in socially anxious groups, particularly as binocular rivalry may represent higher or lower stages of visual processing than other measures. Furthermore, our lack of observed effects may imply that the binocular rivalry differences seen in more severe, independent instances of either condition are not generalizable to comorbid or subclinical cases, which are potentially even more common and should not be overlooked during screening or intervention. Similarly, the lack of observable differences in switch rates between clinical, subclinical, and neurotypical participants suggests the need for further investigations of neural similarities and distinctions between social anxiousness in autism and social anxiety.

## Data availability statement

The original contributions presented in the study are included in the article/Supplementary material, further inquiries can be directed to the corresponding author.

## Ethics statement

The studies involving human participants were reviewed and approved by Brigham Young University Institutional Review Board F19260, F2020-242, and IRB2020-429. The patients/participants provided their written informed consent to participate in this study.

## Author contributions

JO, JM, BB, and JN developed stimulus and experiment design. SK, JO, JM, and HB scheduled participants and administered protocol. HB, JM, JO, and SK processed the data and verified the inter-rater reliability. RL and SK conducted initial literature review and wrote introduction. SK, JO, and HB described methods and materials. SK, JO, and JN developed the analysis script in R and wrote results and conclusions. SK and JN developed script for statistical figures. JO finalized designs, created original methods illustrations, wrote all image captions, and prepared the data files and analysis scripts for sharing on the Open Science Framework. SK and JO wrote the discussion and conclusion. TG and MS verified clinical interpretation of results and provided insight into potential sex differences. RL and JN supervised the manuscript. All authors participated in reviewing, editing, and revising the manuscript.

## Funding

We would like to thank donors for the following funding, without which our research would not be possible: The Honors Office at Brigham Young University (BYU) for funding to SK; Experiential Learning at Brigham Young University for funding to JO; Inter-Disciplinary Research Origination Award program at BYU for funding to JN, TG, MS, and RL; a Mentoring Environment Grant from BYU for funding to RL; and the Burnham Family Foundation for funding to RL.

## Conflict of interest

The authors declare that the research was conducted in the absence of any commercial or financial relationships that could be construed as a potential conflict of interest.

## Publisher’s note

All claims expressed in this article are solely those of the authors and do not necessarily represent those of their affiliated organizations, or those of the publisher, the editors and the reviewers. Any product that may be evaluated in this article, or claim that may be made by its manufacturer, is not guaranteed or endorsed by the publisher.

## Footnotes

^1^We use this term as recommended by many self-advocates we know who prefer the identify-first label “autistic” over person-first terminology “individual with autism spectrum disorder (or condition)” (11).

^2^We use “anxiousness” to refer to the symptoms or traits and “anxiety” to refer to the clinical diagnosis.
