# Supplementary material for: Binocular rivalry in autistic and socially anxious adults
Source: Front Psychiatry. 2023 Jun 28;14:1181797. doi: 10.3389/fpsyt.2023.1181797 (PMC10400451; doi:10.3389/fpsyt.2023.1181797)
Supplement: Supplementary file 1 [file Table_1.docx]

***Supplementary Table 1***

*Outliers Removed by Measure and Diagnostic Group*

| Measure | NT | ASC | SA | Total |
| --- | --- | --- | --- | --- |
| AQ | 0 | 3 | 0 | 3 |
| TCI-HA | 0 | 1 | 0 | 1 |
| RAADS-R | 0 | 0 | 0 | 0 |
| LSAS-Fear | 0 | 0 | 0 | 0 |
| LSAS-Avoidance | 0 | 0 | 0 | 0 |
| LSAS-Total | 0 | 0 | 0 | 0 |
| Spin Average | 1 | 2 | 1 | 4 |
| Neutral Average | 3 | 1 | 0 | 4 |
| Emotional Average | 5 | 1 | 0 | 6 |
| Spin Time 1 | 0 | 0 | 1 | 1 |
| Spin Time 2 | 3 | 2 | 0 | 5 |
| Neutral Time 1 | 2 | 0 | 0 | 2 |
| Neutral Time 2 | 3 | 1 | 0 | 4 |
| Emotional Time 1 | 5 | 1 | 1 | 7 |
| Emotional Time 2 | 3 | 0 | 0 | 3 |
|  |  |  |  |  |

***Supplementary Table 2***

*Average Binocular Rivalry Switch Rates Between Diagnostic Groups*

|  | *pˆ”∗* | *df* | *p* | *p’* |
| --- | --- | --- | --- | --- |
| Spin |  |  |  |  |
| NT vs ASC | 1.415 | 81.16 | 0.1609 | 0.231 |
| SA vs ASC | -0.84359 | 52.026 | 0.4028 | 1.00 |
| NT vs SA | 2.3266 | 36.446 | 0.02566 | 1.00 |
| Neutral |  |  |  |  |
| NT vs ASC | 0.43846 | 64.484 | 0.6625 | 1.00 |
| SA vs ASC | 0.24051 | 61.375 | 0.8107 | 1.00 |
| NT vs SA | -0.0085473 | 32.863 | 0.9932 | 1.00 |
| Emotional |  |  |  |  |
| NT vs ASC | -0.39234 | 68.773 | 0.696 | 1.00 |
| SA vs ASC | 0.037065 | 64.095 | 0.9705 | 1.00 |
| NT vs SA | -0.41101 | 37.946 | 0.6834 | 1.00 |
|  |  |  |  |  |

***Supplementary Table 3***

*Regression Analysis Summary for Self-Report Measure Predicting Switch Rate by Stimulus Condition*

| Spin | | | | | | Neutral | | | | | Emotional | | | | |
| --- | --- | --- | --- | --- | --- | --- | --- | --- | --- | --- | --- | --- | --- | --- | --- |
|  | *β* | *t* | *p* | *p'* | 95% CI | *β* | *t* | *p* | *p'* | 95% CI | *β* | *t* | *p* | *p'* | 95% CI |
| AQ | 0.051 | 0.721 | 0.472 | 1.000 | -6.730, 14.490 | -0.023 | -0.321 | 0.749 | 1.000 | -12.701, 9.149 | -0.063 | -0.901 | 0.369 | 1.000 | -16.237, 6.059 |
| TCI-HA | 0.095 | 1.097 | 0.275 | 1.000 | -11.122, 38.783 | 0.013 | 0.147 | 0.883 | 1.000 | -23.692, 27.502 | -0.025 | -0.293 | 0.770 | 1.000 | -29.996, 22.263 |
| LSAS- Total | 0.257 | 2.031 | 0.048 | 0.630 | 0.764, 138.686 | 0.291 | 2.309 | 0.025 | 0.375 | 10.301, 147.472 | 0.262 | 2.052 | 0.045 | 0.630 | 1.626, 147.023 |
| ADOS-2 | 0.125 | 0.731 | 0.470 | 1.000 | -6.631, 14.037 | 0.239 | 1.471 | 0.151 | 1.000 | -2.449, 15.170 | 0.084 | 0.503 | 0.618 | 1.000 | -7.734,12.805 |

***Supplementary Table 4***

*Group Differences in Binocular Rivalry Switch Rates by Condition and Diagnostic Group*

|  |  | *df* | *SS* | *MS* | *F* | *p* | *p’* |
| --- | --- | --- | --- | --- | --- | --- | --- |
| Model 1 | | 5, 287 |  |  | 20.2 | 2.2e-16^***^ | - |
|  | Condition (All) | 2 | 0.8093 | 0.4047 | 38.900 | 1.12e-15^***^ | - |
|  | Age | 1 | 0.1812 | 0.1812 | 17.419 | 3.98e-05^***^ | - |
|  | Sex | 1 | 0.0576 | 0.0576 | 5.541 | 0.0192^*^ | - |
|  | Medication | 1 | 0.0024 | 0.0024 | 0.235 | 0.6279 | - |
| Model 2 | | 4, 191 |  |  | 5.149 | 0.0005824^***^ | 0.006^*^ |
|  | Condition (Emotional vs. Neutral) | 1 | 0.0022 | 0.00222 | 0.206 | 0.650164 | 1.00 |
|  | Age | 1 | 0.1404 | 0.14041 | 13.076 | 0.000383^***^ | 0.004^*^ |
|  | Sex | 1 | 0.0760 | 0.07596 | 7.074 | 0.008487^**^ | 0.059 |
|  | Medication | 1 | 0.0026 | 0.00259 | 0.241 | 0.623976 | 1.00 |
| Model 3 | | 4, 190 |  |  | 18.83 | 4.659e-13^***^ | <0.0001^***^ |
|  | Condition (Emotional vs. Spin) | 1 | 0.6435 | 0.6435 | 62.807 | 1.88e-13^***^ | <0.0001^***^ |
|  | Age | 1 | 0.0999 | 0.0999 | 9.750 | 0.00207^**^ | 0.017. |
|  | Sex | 1 | 0.0222 | 0.0222 | 2.163 | 0.14306 | 0.715 |
|  | Medication | 1 | 0.0063 | 0.0063 | 0.613 | 0.43447 | 1.00 |
| Model 4 | | 4, 190 |  |  | 17.78 | 2.048e-12^***^ | <0.0001^***^ |
|  | Condition (Neutral vs. Spin) | 1 | 0.5704 | 0.5704 | 55.589 | 3.08e-12^***^ | <0.0001^***^ |
|  | Age | 1 | 0.1239 | 0.1239 | 12.071 | 0.000634^***^ | 0.006^*^ |
|  | Sex | 1 | 0.0268 | 0.0268 | 2.609 | 0.107901 | 0.647 |
|  | Medication | 1 | 0.0087 | 0.0087 | 0.850 | .357768 | 1.00 |
| Model 5 | | 5, 287 |  |  |  | 0.0001099^***^ | - |
|  | Diagnostic Group (All Conditions) | 2 | 0.096 | 0.04781 | 3.714 | 0.025555^*^ | - |
|  | Age | 1 | 0.157 | 0.15657 | 12.163 | 0.000564^***^ | - |
|  | Sex | 1 | 0.069 | 0.06916 | 5.373 | 0.021157^*^ | - |
|  | Medication | 1 | 0.021 | 0.02053 | 1.595 | 0.207613 | - |
| Model 6 | | 5, 91 |  |  | 1.928 | 0.09721 | 0.680 |
|  | Diagnostic Group (Spin) | 2 | 0.0501 | 0.02504 | 2.634 | 0.0773 | 0.627 |
|  | Age | 1 | 0.0382 | 0.03821 | 4.019 | 0.0480^*^ | 0.487 |
|  | Sex | 1 | 0.0016 | 0.00155 | 0.163 | 0.6872 | 1.00 |
|  | Medication | 1 | 0.0018 | 0.00183 | 0.192 | 0.6619 | 1.00 |
| Model 7 | | 5, 92 |  |  | 3.497 | 0.00613 | 0.092 |
|  | Diagnostic Group (Neutral) | 2 | 0.0499 . | 0.02494 | 2.370 | 0.0991 | 0.680 |
|  | Age | 1 | 0.0657 | 0.06570 | 6.245 | 0.0142^*^ | 0.199 |
|  | Sex | 1 | 0.0492 | 0.04917 | 4.674 | 0.0332^*^ | 0.398 |
|  | Medication | 1 | 0.0192 | 0.01921 | 1.826 | 0.1799 | 0.765 |
| Model 8 | | 5, 92 |  |  | 2.383 | 0.0443^*^ | 0.487 |
|  | Diagnostic Group (Neutral) | 2 | 0.0131 | 0.00654 | 0.608 | 0.5465 | 1.00 |
|  | Age | 1 | 0.0565 | 0.05652 | 5.253 | 0.0242^*^ | 0.315 |
|  | Sex | 1 | 0.0362 | 0.03623 | 3.367 | 0.0697 | 0.627 |
|  | Medication | 1 | 0.0223 | 0.02234 | 2.077 | 0.1530 | 0.765 |
|  |  |  |  |  |  |  |  |

Signif. codes:  0 ‘***’ 0.001 ‘**’ 0.01 ‘*’ 0.05 ‘.’ 0.1 ‘ ’ 1

***Supplementary Table 5***

*Group Differences in Binocular Rivalry Switch Rate by Confirmed Diagnosis for Each Stimulus Condition*

|  | *df* | *F* | *SS* | *MS* | *p* | *p’* |
| --- | --- | --- | --- | --- | --- | --- |
| All Conditions | 2 | 0.197 | 0.005 | 0.002581 | 0.821 | - |
| Spin | 2 | 2.173 | 0.0545 | 0.02725 | 0.117 | 0.351 |
| Neutral | 2 | 0.028 | 0.0006 | 0.000315 | 0.972 | 1.00 |
| Emotional | 2 | 0.285 | 0.0061 | 0.003052 | 0.753 | 1.00 |

Signif. codes:  0 ‘***’ 0.001 ‘**’ 0.01 ‘*’ 0.05 ‘.’ 0.1 ‘ ’ 1

***Supplementary Table 6***

*Binocular Rivalry Switch Rates by Diagnostic Group and Condition with Number of Trials*

|  |  | *df* | *SS* | *MS* | *F* | *p* | *p’* |
| --- | --- | --- | --- | --- | --- | --- | --- |
| Model 1 | |  |  |  |  |  |  |
|  | Condition (All) | 2 | 1.147 | 0.5733 | 48.695 | <2e-16^***^ | - |
|  | Number of Completed Trials | 1 | 0.001 | 0.0012 | 0.101 | 0.751 | - |
| Model 2 | |  |  |  |  |  |  |
|  | Condition (Emotional vs. Neutral) | 1 | 0.004 | 0.003926 | 0.343 | 0.558 | 1.00 |
|  | Number of Completed Trials | 1 | 0.000 | 0.000355 | 0.031 | 0.860 | 1.00 |
| Model 3 | |  |  |  |  |  |  |
|  | Condition (Emotional vs. Spin) | 1 | 0.912 | 0.9117 | 76.752 | <2e-16^***^ | <0.0001^***^ |
|  | Number of Completed Trials | 1 | 0.000 | 0.0005 | 0.039 | 0.843 | 1.00 |
| Model 4 | |  |  |  |  |  |  |
|  | Condition (Neutral vs. Spin) | 1 | 0.801 | 0.8014 | 66.624 | 3.56e-15^***^ | <0.0001^***^ |
|  | Number of Completed Trials | 1 | 0.002 | 0.0019 | 0.161 | 0.689 | 1.00 |
| Model 5 | |  |  |  |  |  |  |
|  | Diagnostic Group (All Conditions) | 2 | 0.012 | 0.006000 | 0.444 | 0.642 | - |
|  | Number of Completed Trials | 1 | 0.000 | 0.000286 | 0.021 | 0.884 | - |
| Model 6 | |  |  |  |  |  |  |
|  | Diagnostic Group (Spin) | 2 | 0.0597 | 0.02985 | 2.419 | 0.0914 | 0.548 |
|  | Number of Completed Trials | 1 | 0.0000 | 0.00000 | 0.000 | 0.9971 | 1.00 |
| Model 7 | |  |  |  |  |  |  |
|  | Diagnostic Group (Neutral) | 2 | 0.0043 | 0.002137 | 0.183 | 0.833 | 1.00 |
|  | Number of Completed Trials | 1 | 0.0006 | 0.000581 | 0.050 | 0.824 | 1.00 |
| Model 8 | |  |  |  |  |  |  |
|  | Diagnostic Group (Emotional) | 2 | 0.0035 | 0.001754 | 0.154 | 0.857 | 1.00 |
|  | Number of Completed Trials | 1 | 0.0000 | 0.000033 | 0.003 | 0.957 | 1.00 |

Signif. codes:  0 ‘***’ 0.001 ‘**’ 0.01 ‘*’ 0.05 ‘.’ 0.1 ‘ ’ 1
